# EPHX2 overexpression deters the advancement of clear cell renal cell carcinoma via lipid metabolism reprogramming

**DOI:** 10.3389/fonc.2025.1541429

**Published:** 2025-06-05

**Authors:** Bo Guan, Cong Huang, Jun He, Weimin Shan, Di Cui, Xiaowei Li, Chaozhao Liang, Zongyao Hao

**Affiliations:** ^1^ Department of Urology, Fuyang People’s Hospital, Fuyang, China; ^2^ Department of Urology, the First Affiliated Hospital of Anhui Medical University, Hefei, China; ^3^ Institute of Urology, Anhui Medical University, Hefei, China; ^4^ Anhui Province Key Laboratory of Genitourinary Diseases, Anhui Medical University, Hefei, China; ^5^ Fuyang Medical College, Fuyang Normal University, Fuyang, Anhui, China; ^6^ Department of Nephrology, Fuyang People’s Hospital, Fuyang, China

**Keywords:** ccRCC, EPHX2, lipid metabolism, reprogramming, prognostic marker

## Abstract

**Background:**

Clear cell renal cell carcinoma (ccRCC) is a prevalent and highly aggressive subtype of renal cancer. EPHX2, a metabolic regulator with tumor-suppressive properties, not only influences cell cycle dynamics but also interacts with multiple signaling pathways to inhibit tumor growth. However, the specific mechanistic role of EPHX2 in tumorigenesis remains largely unexplored.

**Objective:**

To elucidate the mechanistic role of EPHX2 in the progression of ccRCC.

**Methods:**

Bulk transcriptome data, single-cell transcriptome data, and gene set data for ccRCC were sourced from the TCGA database and GEO database. An integrated machine learning approach was employed, utilizing the TCGA dataset as the training set and the MTAB dataset as the test set, to develop prognostic models. Subsequently, EPHX2 was stably overexpressed in the 786-O and ACHN cell lines via lentiviral transfection technology, and its functional effects were validated through in vitro experiments.

**Results:**

Through ADIPOGENESIS gene set scoring and differential expression analysis, ten key genes, including EPHX2, were identified. Notably, EPHX2 expression was significantly lower in ccRCC tumor tissues compared to normal renal tissues (P < 0.001). Furthermore, overexpression of EPHX2 significantly inhibited the proliferative, migratory, and invasive capacities of ccRCC cell lines.

**Conclusion:**

EPHX2 plays a pivotal role in the pathophysiological processes of ccRCC, suggesting its potential as a therapeutic target and prognostic biomarker.

## Introduction

Renal cell carcinoma (RCC) arises from the epithelial cells of the renal tubules and ranks among the three principal malignant tumors of the urinary system, being the 14th most prevalent malignancy worldwide (1). In recent years, advancements in molecular biology, genetics, and immunology have greatly enhanced our understanding of the pathogenesis of renal cancer, along with its early diagnosis and treatment, making it a focal point of research both domestically and internationally. Clear cell renal cell carcinoma (ccRCC) is regarded as the predominant subtype of RCC, constituting approximately 70%-80% of all kidney cancer cases (2). Significant progress has been made in the understanding of this disease, establishing ccRCC as a key focus of interest globally. Characteristically, ccRCC possesses a diverse array of tumor tissue components, with a notable feature being the presence of clear cells rich in cytoplasmic content, such as lipid droplets and glycogen deposits (3, 4). These structural variations contribute to the distinct and complex biological behaviors observed in ccRCC, leading to substantial alterations in lipid and glucose metabolism during disease progression (5). This metabolic dysregulation may be intricately linked to tumorigenesis.The occurrence of ccRCC is significantly influenced by the tumor’s biological characteristics. In recent years, there has been a marked increase in the incidence of ccRCC, which is particularly notable for the lack of early symptoms and the variability of clinical presentations. Approximately 25-30% of patients diagnosed with this cancer already exhibit metastasis to other organs (6). Consequently, effectively managing advanced renal malignancies poses a continuous challenge. Renal cancer is responsible for over 100,000 fatalities annually on a global scale (7). recent advancements in molecular imaging, molecular biology, and tumor biology have enhanced our understanding of renal cell carcinoma. Nonetheless, despite the notable progress in diagnostic and therapeutic strategies for ccRCC, the five-year survival rate for patients with metastatic disease remains below 10% (8). The evolution of molecular detection methods has provided insights into cellular proliferative, apoptosis, and metabolic pathways. However, the absence of efficient diagnostic tools and prognostic assessment systems hampers clinicians’ ability to perform early diagnoses and make accurate prognostic predictions for ccRCC patients (9). Thus, the development of effective molecular biomarkers and therapeutic targets is essential for improving patient outcomes (10).

Fatty acids undergo catabolism primarily through fatty acid oxidation (FTO). Peroxidases, which are prevalent in various tissues and organs, play a catalytic role closely tied to lipid peroxidation in cellular membranes. Besides mitochondria, certain fatty acids in animals, such as very long-chain fatty acids (VLCFA) and long branched-chain fatty acids, are oxidized by peroxidases (10). These enzymes catalyze the breakdown of hydrogen peroxide, generating free radicals that are critical in lipid oxidation processes. Peroxidases are pivotal in maintaining the balance of reduction and oxidation within cells. In states of high oxidation, excessive hydrogen peroxide is produced, leading to cellular membrane damage. Consequently, disruptions in lipid metabolism can provoke oxidative stress within cells (11, 12). Therefore, lipid peroxidation-induced damage is an integral component of numerous disease processes, including Parkinson’s disease, diabetes, cancer, and the like.

The EPHX2 gene primarily encodes soluble epoxide hydrolase (SEH), a dual-function enzyme. The N-terminal of SEH exhibits phosphatase activity resulting from the hydrolysis of lipid phosphates, while the C-terminal demonstrates epoxide hydrolase activity, converting epoxides into their corresponding diols (13, 14). This unique dual functionality positions EPHX2 as a key regulator in the complex interplay of lipid metabolism and oxidation. Specifically, the epoxide hydrolase activity of SEH plays a pivotal role in the metabolism of oxidized lipids, where it catalyzes the conversion of reactive epoxide intermediates into less toxic diols. This process is crucial for mitigating the potentially harmful effects of lipid peroxidation products, which can accumulate and contribute to cellular damage if left unchecked. Furthermore, the phosphatase activity of SEH may also indirectly influence lipid oxidation by modulating the availability of lipid phosphates, which are involved in various signaling pathways and membrane dynamics. Studies have shown that certain polymorphisms in the promoter region of the EPHX2 gene may affect the metabolism of lung cancer-related substances by regulating the activity of related enzymes, thereby participating in the occurrence and development of lung cancer. Meanwhile, the study also found that the expression level of the EPHX2 gene was significantly lower in metastatic gastric cancer tissues than in non-metastatic samples. This expression difference may be closely related to the metastasis and invasive capacities of gastric cancer. Further analysis showed that patients with low EPHX2 expression had a significantly shorter survival time than those with high EPHX2 expression, indicating that EPHX2 may be an important indicator for predicting the prognosis of gastric cancer. In addition, the EPHX2 gene also plays a key role in lipid metabolism. Some studies have found that Lactobacillus casei can significantly downregulate the expression of the EPHX2 gene, thus helping to control blood pressure levels. This finding provides new ideas for using probiotics to regulate lipid metabolism. Moreover, through gene knockout mouse experiments, it was found that after the knockout of the EPHX2 gene, there were significant differences in the basic blood glucose and lipid metabolism of mice. Specifically, the blood glucose level of EPHX2 gene knockout mice increased during the glucose tolerance test, and at the same time, significant changes also occurred in serological triglycerides, total cholesterol and other lipid metabolism indicators. These findings further confirm the important role of the EPHX2 gene in glucose and lipid metabolism.

Despite extensive research, the role of the EPHX2 gene in ccRCC remains relatively unexplored. Prior studies have indicated that EPHX2 may possess tumor-suppressive qualities across various cancer types; however, its specific involvement in ccRCC is not yet fully elucidated (15). With the growing comprehension of free radical reactions and lipid peroxidation, researchers are increasingly investigating the role of cyclooxygenase in lipid oxidative injury. Studies examining how EPHX2 influences tumor progression through lipid metabolism reprogramming remain limited. Nonetheless, our bioinformatics analysis identified EPHX2 as a critical gene that impedes the progression of ccRCC. We further predicted its relationship with lipid metabolism and experimentally validated the rationale behind this metabolic model (16).

## Materials and methods

### Data collection

#### ccRCC bulk transcriptome data, single-cell transcriptome data, gene set data acquisition

The bulk transcriptome data and single-cell transcriptome data for ccRCC were sourced from the TCGA database (https://cancergenome.nih.gov/) for comprehensive renal clear cell transcriptome and clinical information. Additionally, the E-MTAB-1980 cohort was retrieved from the ArrayExpress database (https://www.ebi.ac.uk/arrayexpress/experiments/E-MTAB-1980/) for full ccRCC transcriptomic data. The GEO database (https://www.ncbi.nlm.nih.gov/geo/) provided the single-cell sequencing data for clear cell renal cell carcinoma (ccRCC) under the dataset identifier GSE152938. The ADIPOGENESIS gene set was sourced from the MSigDB database (gsea-msigdb.org). We employed the limma package to conduct a differential expression analysis on the transcriptome data from The Cancer Genome Atlas (TCGA), enabling the identification of genes that were significantly up-regulated and down-regulated. Additionally, one-way Cox regression analysis was utilized to filter for pro- and oncogenic genes. Through the GEO database, differential genes were identified using the Robust Rank Aggregation algorithm, which were then intersected with the output from TCGA. A comprehensive importance assessment of the target genes was conducted across the TCGA and MTAB cohorts, leveraging the Gene Importance Assessment Metric for Multiple Cohorts and Machine Learning Algorithms (GMCI Metric) alongside six distinct survival machine learning models. The accuracy of classification obtained served as a weighting coefficient to establish a vector of weights for varied categories, allowing for the computation of a composite score for each sample based on their relative affiliations. Post-screening, genes with non-zero scores were identified and ranked according to their significance, leading to the determination of biologically pertinent key genes in ccRCC.

#### ccRCC single-cell data processing

Processing the ccRCC single-cell data involved analyzing the GSE152938 dataset, which consisted of five samples from four patients, incorporating two ccRCC samples, one papillary renal cell carcinoma sample, one chromophobe renal cell carcinoma sample, and one normal kidney sample. The focus of this study was primarily on ccRCC and normal kidney samples. The raw data were formatted according to the standard 10X file specifications and subsequently organized into Seurat objects for further detailed analysis. During this analytical phase, we filtered cells based on specific criteria: a mitochondrial gene percentage of less than 25%, a hemoglobin gene percentage below 1%, and a ribosomal gene percentage greater than 3%. To mitigate the impact of batch effects, we integrated the data across samples and applied the HARMONY method for correction.

### Prognostic modeling

In terms of prognostic modeling, we designated the TCGA dataset as our training set while using the MTAB dataset as the test set. An integrated machine learning approach was employed to develop prognostic models, which involved ten machine learning algorithms and a total of 101 algorithm combinations. The algorithms utilized included Random Survival Forest (RSF), Elastic Network (Enet), Lasso, Ridge, Stepwise Cox, CoxBoost, Cox Partial Least Squares Regression (plsRcox), Supervised Principal Components (SuperPC), Generalized Augmented Regression Modeling (GBM), and Survival Support Vector Machines (survival-SVM). The signature generation process proceeded through the following steps: (a) univariate Cox regression was applied to identify prognostic mRNAs in the TCGA-ccRCC cohort; (b) a combination of 101 algorithms was executed on the identified prognostic mRNAs to establish predictive models within a leave-one-out cross-validation (LOOCV) framework in the TCGA ccRCC cohort; (c) all models were subsequently evaluated using an external dataset, specifically the E-MTAB-1980 cohort; (d) for each model, Harrell’s consistency index (C-index) was computed across all validation datasets, with the model exhibiting the highest average C-index being regarded as the most efficient.

### Cell culture

786-O and ACHN cells in the renal cancer cell line were purchased from the cell bank of the Chinese Academy of Sciences. Among them, 786-O was cultured on RPMI 1640 medium, while ACHN was cultured on MEM medium. Both different media were supplemented with 10% fetal bovine serum (FBS) as well as 1% penicillin-streptomycin, and then these cells were placed in an incubator containing 37°C and 5% CO2 to promote their continuous growth.

### Lentiviral transfection and reagents

Stable overexpression of EPHX2 was successfully achieved by infecting OE-EPHX2 lentiviral particles into 786-O and ACHN cells (Both the virus and the transfection reagent are from Qingke Biology (Beijing, China), Transfection reagent (TSnanofect, Qingke Biology, Beijing, China). After the cells continued to incubate for 48 h, the transfection rate was detected by fluorescence observation under the microscope, and then the stable strains were screened by continuing the incubation in medium containing 2ug/ml puromycin for 7 days.

### QRT-PCR

Trizol reagent was used to extract the total RNA from the cells. In order to determine the mRNA expression level of the target genes, quantitative real-time PCR (qRT-PCR) was performed using an ABI7500 system with SYBR green fluorescence. The results obtained were compared with the standard curve and were found to be reproducible and stable. qRT-PCR was programmed as follows: from 95°for 30 seconds; from 95°for 5 seconds; from 60°for 34 seconds, with a total of 40 cycles of the test. The reference standard was GAPDH. The order of the primers involved is shown below.

EPHX2: Forward primer 5’-ACTTCGTGCTCGTTCCTCAGATG-3’, Reverse primer 5’-ATTCACCTCGGTTGGCTTGTCC-3’.

GAPDH: Forward primer 5′-CCACCCATGGCAAATTCCATGGCA-3′, Reverse primer 5′-TCTAGACGGCAGGTCAGGTCCACC-3′.

### Western blot

Using RIPA, protease inhibitors, phosphatase inhibitors, and EDTA (50:1:1:1) to lyse cells in whole cell lysate, and using ultrasonic equipment to perform three thorough cell lysations with a 5-minute interval between each lysis. The products of cell lysis will continue to be lysed on ice for 10 minutes. Then, we use centrifugation to collect the products after cracking. Finally, we measured the concentration of protein, separated the same amount of protein using 10% SDS-PAGE gel, and then transferred it to PVDF membrane. After blocking with 5% skim milk for 2 hours, the membrane was incubated overnight with EPHX2 antibody (derived from Proteintech Group Cat No. 10833-1-AP) on a 4° shaker. Wash the membrane three times with TBST, then incubate the secondary antibody at room temperature for 1 hour, and wash the membrane again three times. Finally, ECL reagent was used for exposure and development treatment.

### CCK-8 proliferative assay

Five 96-well plates were inoculated with 100 uL of cell suspension (approximately 2000 cells) in consecutive 96-well plates. Next, a 24-hour pre-culture was performed at 37°C,5% CO2 in an incubation environment. In each condition, three wells were repeated. Next, one 96-well plate was removed each day and 10 uL of cck-8 reagent was added dropwise to each well, and then incubation was continued in the incubator for 1 hour. Next, we measured the absorbance of each well at 450 nm using an enzyme marker. In this way, we assessed the growth activity of the cells and their growth status.

### Transwell infestation detection

We used polycarbonate transwell filters as experimental materials. Matrigel gel was diluted with serum-free cell culture medium at a ratio of 1:8 at 4°C. 100μL of the diluted matrix gel mixture was aspirated and added vertically into the upper chamber of the Transwell, evenly flattened on the bottom, and placed in the incubator at 37°C for 3h. to allow for the synthesis of the gel. After incubation, 100uL of serum-free medium was added to each well and continued to be placed in the incubator at 37°C for 30 min for basement membrane hydration. The upper chamber of the 24-well plate was inoculated with 786-O as well as ACHN cells at a concentration of 2000/uL, respectively (3 replicate wells were set up for each cell line in each group). Then 500 uL of medium containing 10% FBS was added to the lower chamber. The cells were then incubated in a 5% CO2 incubator at 37°C for 48 h. Finally, we fixed the invaded cells with 4% paraformaldehyde and stained them with crystal violet, and then observed the stained cells under an inverted microscope and analyzed the results using Image J software.

### Wound healing assay

Before inoculating the cells. Four parallel lines were drawn with a ruler to mark the back of the six-well plate at equal distances, respectively. Subsequently, 786-O and ACHN cell lines were inoculated into the six-well plate and cultured for 24 h. When the confluent density of cells reached 95%, the cells were scraped with a 200 uL lance tip vertically in the six-well plate to form a vertical wound. The scraped cells were then washed with PBS, fresh medium was added to continue the culture in the incubator, and images of cell migratory were obtained by inverted microscope after 0H and 24H. Finally, the width of the scratch was measured by Image J software and the results were analyzed.

### Validation through tissue microarray

To comprehensively examine the expression alterations of EPHX2 within cancerous tissues, we collaborated with Qingdao Intelligent Biotechnology Co. This organization conducted immunohistochemical tissue microarray experiments utilizing tissue microarrays that we supplied. Details regarding the antibody employed: EPHX2 antibody was sourced from Proteintech Group, catalog number 10833-1-AP.

### Statistical analysis

All database analyses were executed using R software (version 4.2.2). In this investigation, the statistical characteristics of the dataset were further explored through an experimentally validated framework. The Metascape database was utilized for functional annotation (https://metascape.org), while the ggplot2 package in R was employed for visual representation. The findings of this study were corroborated through data processing and interpretation to confirm alignment with the anticipated outcomes. Experimental data were analyzed using GraphPad Prism 9 software, and each experiment was replicated independently three times. A p-value threshold of <0.05 was utilized to determine statistical significance.

## Results

### Key gene screening in ADIPOGENESIS

**Figure 1:**
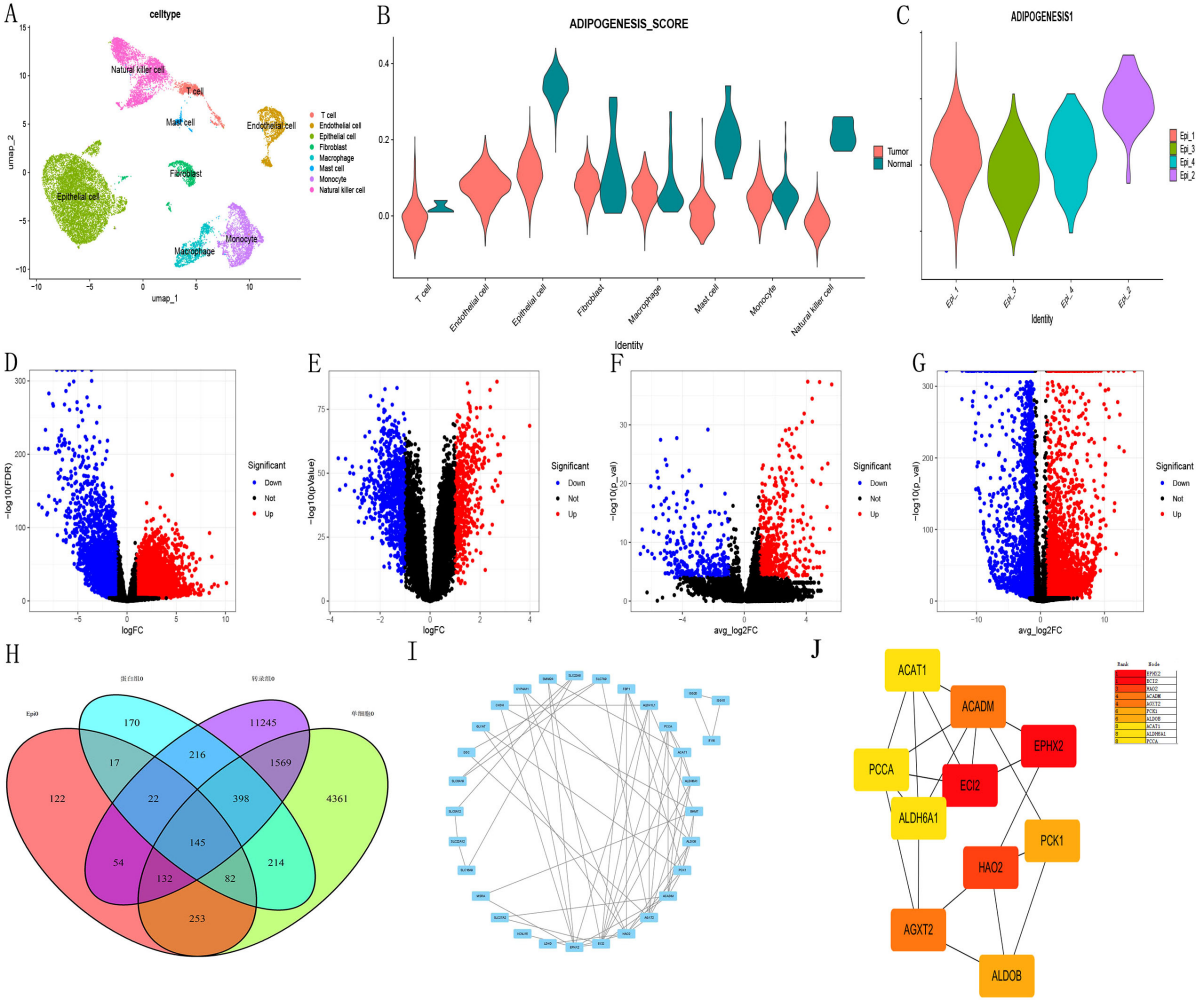
Illustrates the key gene screening for ADIPOGENESIS. **(A)** single-cell data analysis, **(B)** scoring differences in adipogenesis-related gene sets across cell subtypes, **(C)** Identification of four tumor epithelial cell subtypes (Ep1–Ep4) with notable scoring disparities between Ep2 and Ep3. **(D)** Differential gene expression analysis of transcriptomic datasets sourced from The Cancer Genome Atlas (TCGA) database for clear cell renal cell carcinoma (ccRCC). **(E)** Proteomic data for ccRCC obtained from Clinical Proteomic Tumor Analysis Consortium (CPTAC) database. **(F)** Analysis revealed differences in single-cell RNA data when comparing tumor cells to epithelial cells. **(G)** Transcriptomic profiling identified 827 distinct RNA species in tumor epithelial subtypes Ep2 and Ep3. **(H)** Intersection of four protein/transcriptomic datasets revealed 145 differentially expressed genes. **(I)** Protein-protein interaction (PPI) network analysis via STRING database identified 40 adipogenesis-associated genes. **(J)** Top 10 hub genes selected by degree algorithm using CytoHubba plugin in Cytoscape software.

Initially, we conducted a rigorous series of quality control measures, filtering, and dimensionality reduction clustering on the single-cell dataset (**Figure 1A**). Subsequently, we employed the ADIPOGENESIS gene set to score various cell subtypes. The results revealed marked differences in ADIPOGENESIS scoring between tumor epithelial cells and normal renal epithelial cells (**Figure 1B**). Further clustering analysis categorized tumor epithelial cells into four distinct subtypes (Ep1-4), each assigned specific ADIPOGENESIS scores. Notably, the most significant disparity in ADIPOGENESIS scores was observed between Ep2 and Ep3 (**Figure 1C**). To pinpoint core genes associated with ADIPOGENESIS in clear cell carcinoma, we processed renal clear cell carcinoma transcriptome data from TCGA, successfully identifying 13,781 differentially expressed RNAs between normal and tumor samples (**Figure 1D**) (P<0.0001, FC>1). Additionally, proteomic analysis from CPTAC revealed 1,264 differential proteins between normal and tumor states (**Figure 1E**) (P<0.0001, FC>1). The single-cell data highlighted 7,154 differentially expressed RNAs between normal and tumor epithelial cells (**Figure 1F**) (P<0.0001, FC>1) and 827 differentially expressed RNAs between Ep2 and Ep3 (**Figure 1G**) (P<0.0001, FC>1). Ultimately, the intersection of these four differential protein and transcriptome datasets yielded 145 differential genes (**Figure 1H**). When combined with TCGA survival data, 40 genes exhibited significant associations with univariate prognosis (P <0.0001) and 40 ADIPOGENESIS-related genes were identified within the protein interaction network on the STRING website (**Figure 1I**). Further analysis via degree analysis in Cytoscape software identified the top 10 genes (EPHX2, ECI2, HAO2, ACADM, AGXT2, PCK1, ALDOB, ACAT1, ALDH6A1, PCCA) derived from cytoHubba assessment based on degree (**Figure 1J**).

ADIPOGENESIS Key Model Construction.

### Adipogenesis-related gene key model construction

**Figure 2:**
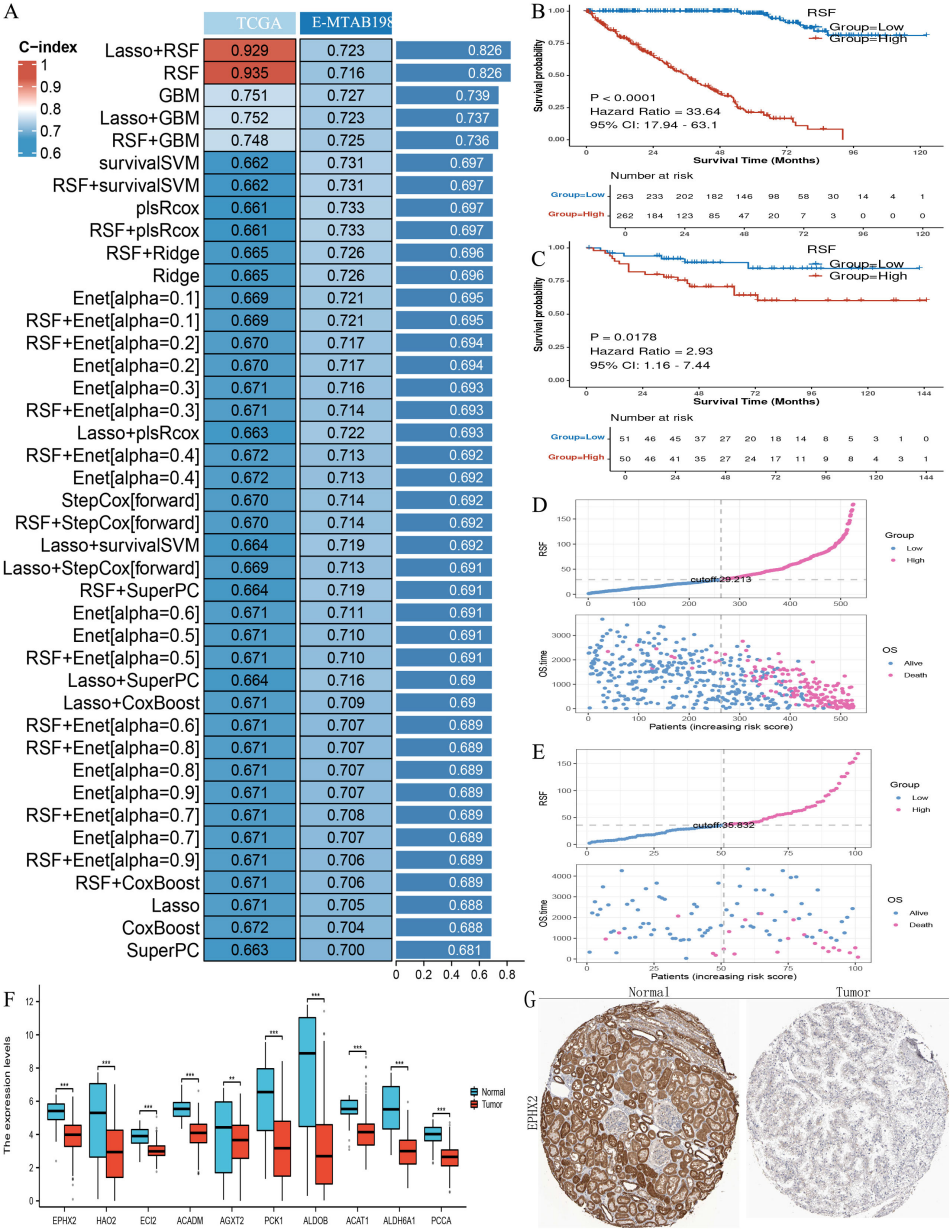
Illustrates the construction of prognostic models using the ten key adipogenesis (ADIPOGENESIS)-related genes. **(A)** Construction of a prognostic model evaluated by the concordance index (C-index) based on the ten adipogenesis-related genes. **(B)** Kaplan-Meier (KM) survival analysis in the training cohort (TCGA-KIRC). **(C)** KM survival analysis in the validation cohort (E-MTAB-198, ArrayExpress). **(D)** Risk factor correlation analysis in the TCGA-KIRC cohort. **(E)** Risk factor association analysis in the E-MTAB-198 cohort. **(F)** Differential expression of the ten key genes in TCGA clear cell renal cell carcinoma (ccRCC) (tumor vs. normal). **(G)** Immunohistochemical (IHC) staining of EPHX2 in normal renal tissues and ccRCC tumor tissues (sourced from the Human Protein Atlas [HPA]), demonstrating downregulation of EPHX2 in ccRCC.

In an effort to explore the prognostic implications of the ten genes related to ADIPOGENESIS in ccRCC, we developed a prognostic model utilizing two extensive ccRCC datasets (namely, the TCGA-KIRC dataset and the E-MTAB-198 dataset). This model was constructed based on the ten identified genes, and we subsequently validated its prognostic predictive capacity. Our findings indicated that the Random Survival Forest (RSF) models for these ten genes exhibited considerable efficacy in prognostic evaluation, achieving an accuracy rate of 0.935 for the TCGA dataset and 0.716 for the E-MTAB-198 dataset (**Figures 2A-G**).

### EPHX2 expression is decreased in human CCRCC tissues

**Figure 3:**
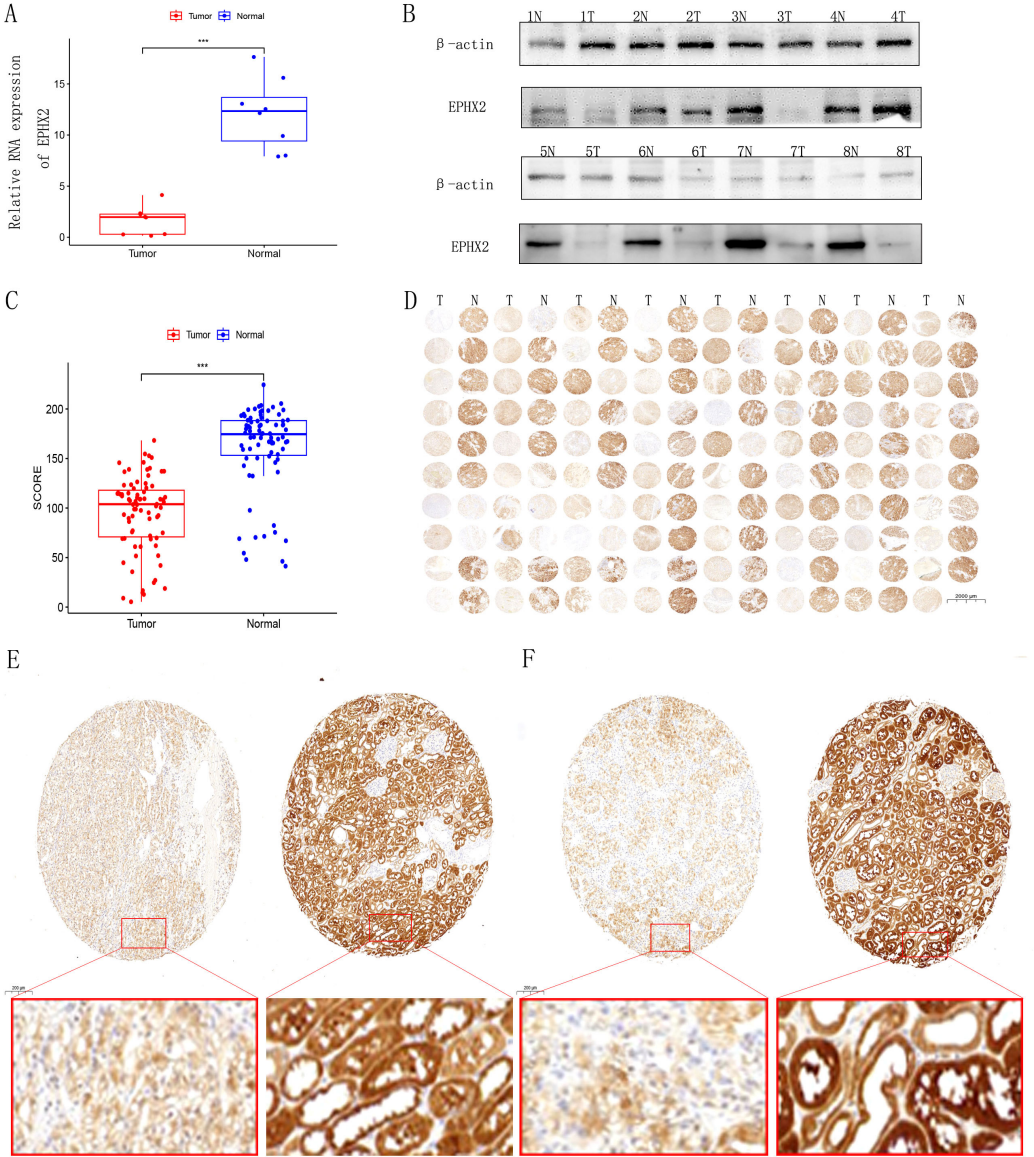
Expression analysis of EPHX2 in human clear cell renal cell carcinoma (ccRCC). **(A)** Relative EPHX2 mRNA expression in tumor tissues compared to paired normal renal tissues (n = 8 ccRCC patients; P < 0.001). **(B)** Western blot analysis of EPHX2 protein levels in tumor and paired normal tissues (n = 8 ccRCC patients). **(C, D)** Immunohistochemical (IHC) staining of EPHX2 in tumor and normal renal tissues (n = 80 ccRCC patients). **(E, F)** Comparative quantitative IHC analysis of EPHX2 expression in tumor tissues versus matched normal kidney tissues (n = 80; P < 0.001).

The analysis identified ten key ADIPOGENESIS-related genes (EPHX2, ECI2, HAO2, ACADM, AGXT2, PCK1, ALDOB, ACAT1, ALDH6A1, PCCA), with EPHX2 ranking first based on DEGREE analysis. To confirm the differential expression of EPHX2 between tumor and normal kidney tissues, we conducted quantitative PCR (qPCR), western blotting (WB), and histological microarray validations. Initial assessments involved eight pairs of fresh tissue specimens from normal and ccRCC patients for qPCR and WB validation, revealing that both RNA and protein levels of EPHX2 were significantly reduced in tumor samples (**Figures 3A, B**). Further validation through tissue microarray confirmed these findings (**Figures 3C-F**).

### High expression of EPHX2 may be a prognostic biomarker for ccRCC patients

**Figure 4:**
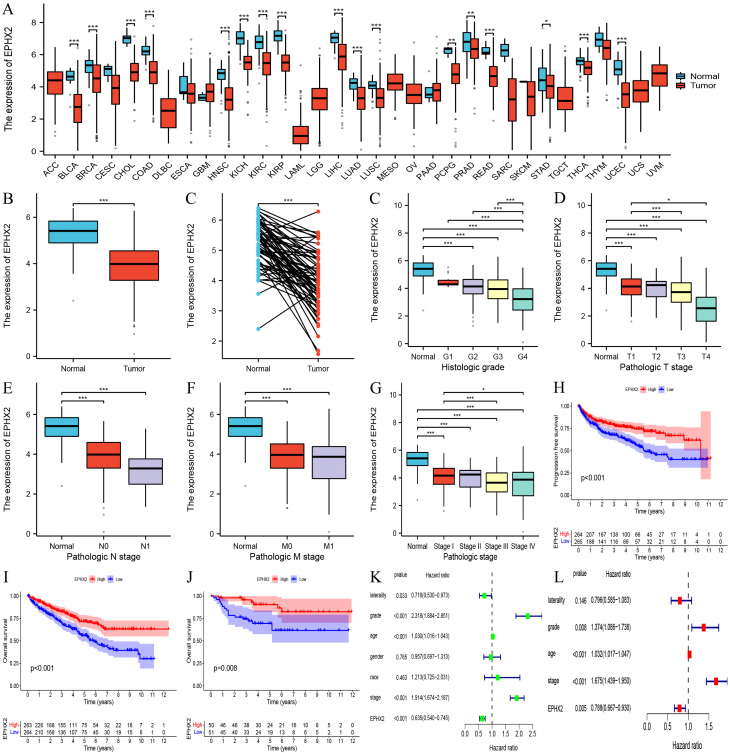
Association of EPHX2 expression with clinical characteristics and prognosis in ccRCC. **(A)** Pan-cancer EPHX2 mRNA expression across tumor types in the TCGA database. **(B)** Differential EPHX2 expression between ccRCC tumors (n = [X]) and normal kidney tissues (n = [X]) in the TCGA-KIRC cohort ([P-value]). **(C)** EPHX2 expression in paired tumor-normal samples from ccRCC patients (n = [X]; [P-value]). **(D)** Correlation between EPHX2 expression and tumor grade in ccRCC. **(E)** Association of EPHX2 expression with N-stage (lymph node metastasis). **(F)** Association of EPHX2 expression with M-stage (distant metastasis). **(G, H)** Kaplan-Meier survival analysis of EPHX2 for **(G)** progression-free survival (PFS) and **(H)** overall survival (OS) in the TCGA-KIRC cohort (P = [value]). **(I)** Validation of EPHX2 prognostic significance for OS in the independent E-MTAB-198 cohort (P = [value]). **(J)** Univariate analysis of EPHX2 association with clinicopathological parameters in TCGA. **(K, L)** Multivariate Cox regression analysis of EPHX2 in relation to clinical characteristics in TCGA data.

Our findings suggest that elevated expression levels of EPHX2 may serve as a prognostic biomarker for patients with ccRCC. An in-depth analysis of the EPHX2 gene, which ranked highest by DEGREE, was conducted. We examined EPHX2 expression across 33 human cancers and normal tissues utilizing the TCGA database. The results demonstrated a significant disparity in EPHX2 expression within ccRCC tissues relative to other tumor types, with normal tissues exhibiting higher expression levels (**Figures 4A, B**). To further substantiate the differential expression of EPHX2 in ccRCC, we successfully constructed a data model representing the ccRCC tumor and normal groups, facilitating pairwise difference analysis via the TCGA database (**Figure 4C**). Understanding the correlations between molecular markers and cancer progression is essential for enhancing our comprehension of disease dynamics and clinical management. Hence, we leveraged the TCGA database to further investigate these relationships. The variations in EPHX2 expression across different TNM staging classifications, as well as the corresponding prognostic implications and clinical characteristics in clear cell renal cell carcinoma (ccRCC) tumors, are illustrated in (**Figures 4D-L**).

### CCRCC cell migratory and invasive capacity is inhibited by EPHX2 overexpression

**Figure 5:**
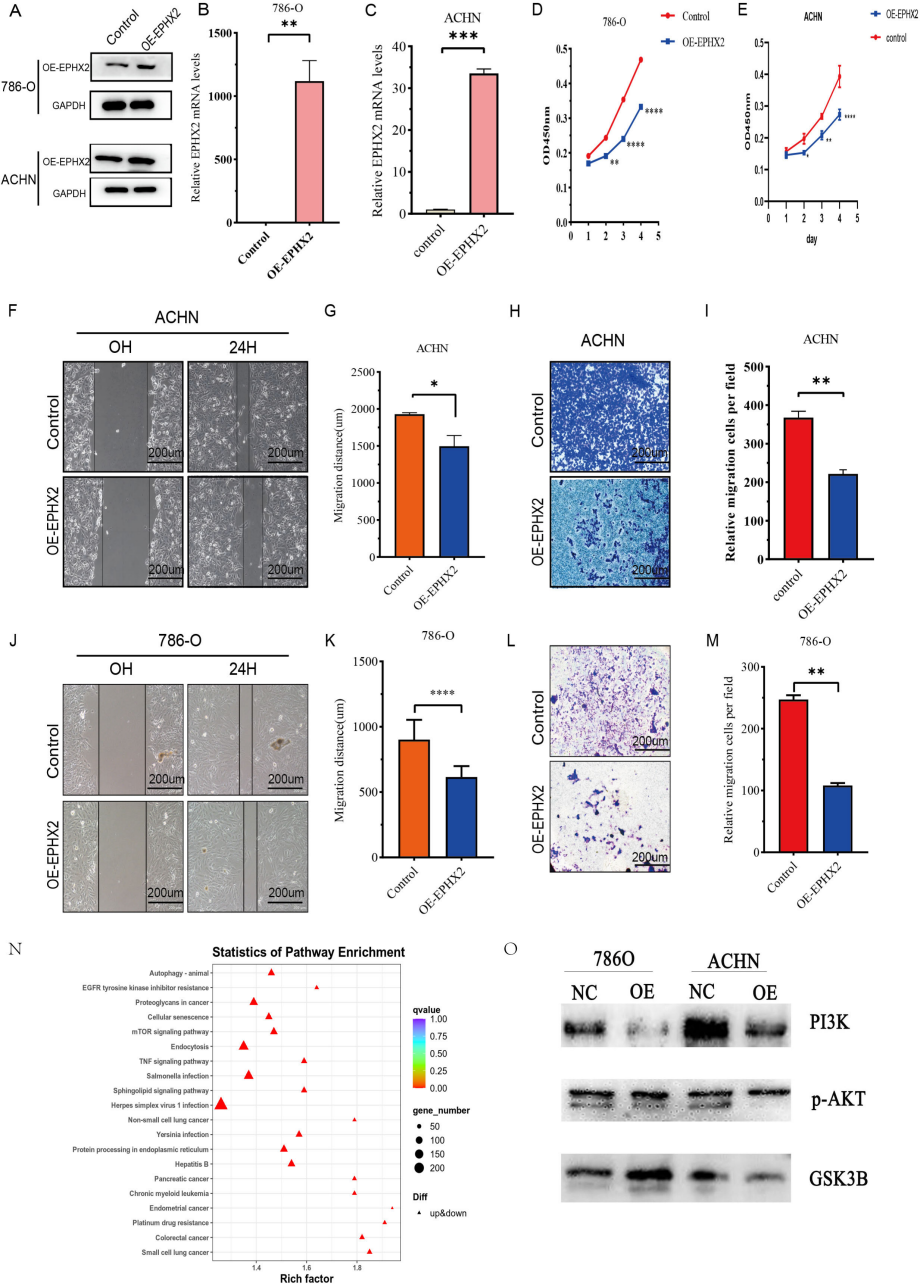
illustrates the association of EPHX2 expression levels with the migratory and invasive properties of ccRCC cell lines. **(A)** presents a representative quantitative analysis of EPHX2 protein levels in two distinct ccRCC cell lines, 786-O and ACHN, utilizing western blotting protein method. **(B, C)** display the results of quantitative reverse transcription-polymerase chain reaction (qRT-PCR) performed on 786-O and ACHN cell lines, respectively. These experiments were designed to evaluate the transfection efficiency of EPHX2-targeting constructs or control vectors, ensuring that any observed changes in cellular behavior could be attributed to alterations in EPHX2 expression rather than variations in transfection efficacy. **(D, E)** depict the absorbance at 450 nm (OD450 nm), measured using the Cell Counting Kit-8 (CCK-8) reagent, at specified time intervals post-transfection. This assay was employed to assess cell viability and proliferation rates, allowing for the investigation of potential growth advantages or disadvantages conferred by altered EPHX2 expression. **(F, J)** illustrate the results of the wound healing assay conducted on 786-O and ACHN cells, respectively, at 0 and 24 hours post-wounding in both experimental and control groups. **(G, K)** quantitatively analyze the wound healing assay results using Image J software image analysis tool. **(H, L)** report on transwell assays performed to assess the invasive capacity of 786-O and ACHN cells after 24 hours of incubation. In these assays, cells are plated on a porous membrane coated with extracellular matrix components, and their ability to invade through the membrane is quantified. **(I, M)** provide the quantitative analysis of the transwell assay results using Image J software. Lastly, **(N, O)** presents single-cell sequencing outcomes for the ACHN cell lines. The scale bar in panels **(F, H, J, L)** is set at 200 µm, and all experiments were conducted independently in triplicate (noted as * p < 0.05, ** p < 0.005, *** p < 0.001, **** p < 0.0001).

The overexpression of EPHX2 significantly hinders the migratory and invasion capabilities of ccRCC cells. To evaluate the biological implications of EPHX2 in the progression of ccRCC, our research team employed 786-O and ACHN cell lines to establish lentiviral transfection models. These models were successfully validated through quantitative real-time PCR (qRT-PCR) as shown in (**Figures 5B, C**). Additionally, Western blot analysis corroborated the transfection efficacy in both cell lines (**Figure 5A**). In order to assess the proliferative capacity of ccRCC cells post-transfection, we utilized a CCK-8 assay on 786-O and ACHN cells. The outcomes revealed that the overexpression of OE-EPHX2 led to a statistically significant difference from the control group as early as 24 hours post-transfection (**Figures 5D, E**). Moreover, wound healing assays indicated that the migratory capability of both 786-O and ACHN cells was markedly reduced in the OE-EPHX2 experimental group compared to controls (**Figures 5F, G, J, K**). In parallel, transwell assays demonstrated that the control group exhibited markedly enhanced invasive abilities relative to the experimental group (**Figures 5H, I, L, M**). Collectively, these findings suggest that EPHX2 overexpression substantially impedes the proliferative, migratory, and invasion of ccRCC cells. To further elucidate the regulatory mechanisms by which EPHX2 influences ccRCC tumors following overexpression, we conducted single-cell transcriptome sequencing on ACHN cells transfected with the EPHX2 gene (**Figures 5N, O**).

## Discussion

In summary, as one of the three predominant malignant tumors affecting the urinary system, the incidence of renal cell carcinoma continues to be a significant concern. RCC has been on the rise globally, with clear indications that ccRCC constitutes approximately 80% of all RCC cases. Despite notable advancements in medical technologies—particularly in molecular biology, genetics, and immunology—significant challenges persist in the management of ccRCC, primarily due to its highly aggressive nature and dismal prognosis. Evidence suggests that ccRCC exhibits resistance to conventional chemotherapy, radiotherapy, and targeted treatment modalities, resulting in recurrence rates and a five-year survival rate of approximately 40% and 20%, respectively, post radical nephrectomy. Consequently, there is an urgent need for further research into the mechanisms underlying tumorigenesis and metastasis in ccRCC, which could lead to the identification of reliable and actionable biomarkers (17).

Fatty acids are pivotal in cellular metabolism, contributing to the regulation of membrane synthesis and its fluidity. Additionally, they function as second messengers in signaling pathways that help maintain homeostasis and serve as energy reserves in animal tissues (18, 19). Alterations in lipid metabolism are among the most significant metabolic adaptations observed in tumors, necessitated by the requirement to sustain redox homeostasis during tumor cell activities (20, 21). EPHX2 encodes soluble epoxide hydrolase (SEH), a crucial enzyme that influences various metabolites by facilitating the catabolism of epoxidized fatty acids into corresponding diols, thereby modulating lipid signaling functions (22, 23). The activity of SEH can diminish lipid signaling effects (24). Moreover, studies have indicated that inhibitors of EPHX2 can accelerate the progression of melanoma and fibrosarcoma in murine models by augmenting levels of the endogenous lipid mediator epoxyeicosatrienoic acid (EET), further implicating EPHX2 in lipid metabolism and tumor progression (25).

In our investigation, we concentrated on the EPHX2 gene’s involvement in ccRCC, conducting a thorough analysis of its biological role and its potential as a prognostic biomarker. Downregulation of EPHX2 expression leads to decreased SEH activity, subsequently reducing the degradation of oxidized lipids. This may result in the accumulation of lipid peroxidation products, which are highly cytotoxic and capable of damaging cell membranes, DNA, and proteins, thereby promoting tumor initiation and progression (1, 26). Notably, lipid peroxides are not only a source of cellular cytotoxicity but may also act as signaling molecules involved in tumor cell proliferation, migration, and invasion. Downregulation of EPHX2 may promote malignant behaviors of tumor cells by affecting lipid signaling pathways, such as the phosphatidylinositol-3-kinase (PI3K)/protein kinase B (AKT) signaling pathway. Studies have demonstrated that EPHX2 inhibits tumor progression in various cancers by regulating lipid metabolism, supporting its analogous role in ccRCC. Additionally, lipid metabolic reprogramming provides tumor cells with abundant energy and biosynthetic precursors, supporting their rapid proliferation. Downregulation of EPHX2 may accelerate ccRCC progression by promoting a shift in lipid metabolism toward a state that favors tumor cell proliferation. Our findings revealed a marked reduction in EPHX2 expression within tumor tissues, corroborated by data mining efforts and the analysis of human ccRCC tissue samples. Notably, levels of EPHX2 exhibited a significant correlation with both the clinical stage and histological grade of patients, indicating its critical role in ccRCC pathogenesis. By transfecting lentiviruses that overexpress EPHX2 into ccRCC cell lines, we were able to validate results from bioinformatics analyses, demonstrating that EPHX2 overexpression effectively inhibited cellular proliferative, migratory, and invasion (27).

These findings support the hypothesis that EPHX2 could serve as a promising biomarker for the diagnosis and therapeutic intervention of ccRCC. Beyond its implications on tumor biology, the association between EPHX2 expression and patient prognosis highlights its potential utility as a prognostic marker for ccRCC. The inverse relationship between diminished EPHX2 levels and advanced tumor stages accentuates the necessity for identifying molecular markers that can guide clinical decision-making and therapeutic strategies. Importantly, leveraging machine learning models to evaluate EPHX2 alongside other prognostic factors could enhance the precision of patient stratification, thereby fostering a more tailored treatment approach. This is especially critical in light of the recognized heterogeneity of ccRCC and the pressing need for targeted therapies that accommodate individual tumor profiles and genetic predispositions (28, 29). Although this study aimed to deeply explore the potential mechanism of EPHX2 in ccRCC by constructing machine learning models and prognostic models, based on the existing data and methods, we recognize that there are several limitations in the current analytical framework, which may affect the comprehensiveness and accuracy of the research conclusions. Firstly, due to the limitation of sample size, the dataset in this study is relatively small. This situation may restrict the stability and reliability of the model prediction accuracy. When the sample size is insufficient, machine learning models may have difficulty fully capturing the true distribution characteristics inherent in the data, thereby increasing the risk of overfitting or underfitting and resulting in limited generalization ability of the model. Secondly, the interpretability of the Kaplan-Meier survival analysis results highly depends on the rationality of patient grouping. Specifically, in this study, the grouping strategy based on the expression level of EPHX2 was adopted, and the choice of the grouping threshold has a significant impact on the shape of the survival curve and the final conclusions. Different grouping criteria may lead to completely different survival analysis results. This uncertainty indicates that we need to further optimize the grouping strategy in subsequent studies to enhance the robustness of the conclusions. Furthermore, although this study used the E-MTAB-1980 dataset as an external validation set to evaluate the external validity of the model, the sample size and diversity of this validation set are still insufficient. This limitation may restrict the assessment of the model’s applicability in a broader clinical context and affect our comprehensive understanding of the model’s generalization ability. Finally, in the process of model evaluation in this study, we mainly focused on single indicators such as survival analysis and prediction accuracy, while relatively neglecting the consideration of key performance dimensions such as model robustness, stability, and interpretability. A single evaluation indicator may not comprehensively reflect the comprehensive performance of the model and its potential value in actual clinical applications. Therefore, future research needs to introduce a multi-dimensional evaluation system to more comprehensively evaluate the model’s performance. Although this study provides preliminary insights into the role of EPHX2 in ccRCC, future research still needs to make continuous efforts in expanding the sample size, optimizing the grouping strategy, enhancing external validation, and improving the evaluation system, with the aim of more accurately revealing the specific mechanism of EPHX2 in the occurrence and development of ccRCC.

## Conclusion

In conclusion, the limitations inherent in this study warrant careful scrutiny. Primarily, the relatively modest sample size, particularly concerning cellular and clinical specimens, may restrict the broader applicability of our findings. This limitation is particularly significant in the context of translational research, where larger cohorts are often essential to validate results. Validation of the implications derived from biomarker studies is frequently necessary. Additionally, the potential batch effect inherent in high-throughput analyses may introduce variability that could obscure the findings, thereby compromising the reliability of the established associations. Moreover, the absence of long-term follow-up data concerning patient prognosis restricts the capacity to arrive at conclusive insights regarding the prognostic value of EPHX2 in ccRCC. It is imperative that future research addresses these limitations to enhance the dependability of EPHX2 as a biomarker and to elucidate its involvement in the molecular pathogenesis of renal cell carcinoma.

The current study highlights the pivotal role of EPHX2 in the pathophysiology of renal clear cell carcinoma, indicating its promise as a therapeutic target and prognostic biomarker. The observed downregulation of EPHX2 in tumor tissues correlates with aggressive tumor characteristics and unfavorable patient outcomes, underscoring the necessity for further exploration of its mechanistic pathways, particularly concerning the PI3K/AKT signaling pathway. The integration of bioinformatics methodologies with experimental validation fosters the development of novel therapeutic strategies and comprehensive prognostic models. Future investigations should strive to overcome existing constraints by concentrating on larger patient cohorts and extended follow-up periods to validate the clinical significance of EPHX2 in the management of ccRCC.

## Data Availability

All relevant data is contained within the article. The original contributions presented in the study are included in the supplementary material, further inquiries can be directed to the corresponding authors.
